# Testicular adrenal rest tumors in boys with 21-hydroxylase deficiency, timely diagnosis and follow-up

**DOI:** 10.1530/EC-18-0097

**Published:** 2018-03-12

**Authors:** Mirjana Kocova, Vesna Janevska, Violeta Anastasovska

**Affiliations:** 1 Department of Endocrinology and GeneticsMedical Faculty, University Pediatric Clinic, Ss. Cyril and Methodius University, Skopje, Republic of Macedonia; 2 Institute of PathologyMedical Faculty, Ss. Cyril and Methodius University, Skopje, Republic of Macedonia; 3 Genetic LaboratoryMedical Faculty, University Pediatric Clinic, Ss. Cyril and Methodius University, Skopje, Republic of Macedonia

**Keywords:** 21-hydroxylase deficiency, *CYP21A2* gene, metabolic control, testicular adrenal rest tumors, ultrasound

## Abstract

**Background:**

Testicular adrenal rest tumors (TARTs) are found in 30–94% of adult males with congenital adrenal hyperplasia (CAH). We sought to explore TART appearance through yearly ultrasound examination of testes in young boys with CAH, and its association with metabolic control and genetic mutations.

**Methods:**

Twenty-five boys with 21-hydroxylase deficiency in the age group 4–18 years diagnosed during the period 2001–2016 were included in the study. ACTH, 17-hydroxyprogesterone, androstenedione and testosterone were measured at 4-month intervals. Growth and BMI were assessed at the time of evaluation. PCR/ACRS method was used for *CYP21A2* gene analysis. Testicular ultrasound examination was performed yearly.

**Results:**

TARTs were detected by ultrasound in 8 children at the age of 6–16 years (13.2 years average). Five had salt-wasting form, two had simple virilizing form and one had non-classic form of CAH. Significant differences in the17OHP and androstenedione levels were detected between the boys, adherent and non-adherent to therapy. Inadequate metabolic control was not different in boys with and without TART (11/17 and 5/8 respectively). No significant difference was detected in the distribution of genetic mutations or adherence to therapy between patients with and without TARTs. One patient had a mutation not reported thus far in TART and another developed leukemia.

**Conclusion:**

TART is not rare in young boys with CAH, irrespective of the specific mutation or metabolic control. Ultrasound screening helps timely diagnosis and adjustment of therapy.

## Introduction

Congenital adrenal hyperplasia (CAH) is most frequently caused by deficiency of 21-hydroxylase, a key enzyme in the mineralocorticoid and glucocorticoid synthesis pathways in humans. It is a rare autosomal recessive disease with a frequency of 1:15,000 (1, 2). However, heterozygosity is frequent in this disease, especially in certain populations (3). The clinical forms of CAH are classified as classical and non-classical. The classical forms are the salt wasting (SW) form (2/3 of patients) and simple virilizing (SV) form (~1/3 of patients), requiring long-standing therapy with hydrocortisone and fludrocortisone and careful titration to avoid overdosing. Major complications of the disease and the therapy are shorter than expected stature, obesity, metabolic syndrome and infertility (1, 2, 4). Fertility problems in adult male patients with CAH have been attributed to the development of testicular adrenal rest tumors (TARTs) (3, 4, 5). The migration of adrenal cells during embryonic development can involve extra-adrenal regions, such as the retroperitoneum, ovaries and testes. These adrenal cells undergo atrophy later during the development if the hormonal status of the individual is normal. However, if the adrenocorticotropic hormone (ACTH) level in the serum is high, as is frequently the case in patients with CAH, these ‘ectopic’ adrenal cells might multiply to a level of severe tumor-like hyperplasia affecting the testicular tissue and causing infertility later in life (5, 6). TART lesions were first described as early as 1940, but the risk factors, genetic associations and optimal therapy are still under debate. Most studies assessing the incidence of TART include adult patients or both children and adults. The usual age at diagnosis is 20–40 years with a variable prevalence reaching up to 94% (4, 5, 6, 7). Some recent studies show that it can also affect younger boys (8). Severe *CYP21A2* gene mutations, late diagnosis, non-compliance and difficulties in managing CAH, e.g. maintaining ACTH values within acceptable levels can increase the likelihood for TART (8, 9, 10, 11). However, these findings have been disputed (12, 13). No international guidelines for follow-up of male children with CAH and TART are available. New ENDO guidelines for CAH are in a process of comment before publication.

Here, we describe the experiences of a single center treating children with CAH associated with TART. The detection of TART relied on yearly testicular ultrasonography check-ups starting at the age of 4 years. The study covers a 15-year follow-up period. These findings increase the limited data on TART in children and contribute to the assessment of the diagnostic value of ultrasound and possibly to the development of follow-up algorithms.

## Subjects and methods

During the period 1991–2015, 71 patients (30 males) with all forms of CAH were diagnosed at the University Pediatric Clinic in Skopje, covering the entire pediatric tertiary care of the Republic of Macedonia. Written consent for yearly testicular ultrasonographic check-up was obtained by the parents of each patient. The study was conducted according to the declaration of Helsinki, revision of 2008. It was approved by the Ethical committee at the University Pediatric Clinic.

The diagnosis of CAH was based on the international guidelines (14). Testicular enlargement was detected in only one boy. Molecular analysis was performed using the polymerase chain reaction/amplification creation restriction site (PCR/ACRS) method for detection of the nine most frequent point mutations (p.P30L (c.92C>T); In2G (c.293-13A/C>G); p.G110Efs (c.332_339del); p.I172N (c.518T>A); exon 6 cluster mutations: p.I236N (c.710T>A), p.V237E (c.713T>A), p.M239K (c.719T>A); p.V281L (c.844G>T); p.Leu307fs (c.923_924insT); p.Q318X (c.955C>T) and p.R356W (c.1069C>T)) in the *CYP21A2* gene. Subsequent restriction analysis, visualized on 2% agarose gel electrophoresis using ethidium bromide staining allowed detection and determination of the zygosity of the mutation, as described previously (15). All patients received oral therapy with hydrocortisone in a total dose 10–25 mg/m^2^/day divided in 3 daily doses. In patients with the SW form of the disease fludrocortisone (0.05–0.2 mg divided in 3 doses per day) was given orally prior to puberty. Therapy was titrated aiming to achieve a 17-OH progesterone (17-OHP) level below 10 ng/mL. Follow-ups were scheduled at 4-month intervals and included measurement of the height and weight (BMI), pubertal stage, testis volume and the levels of electrolytes, 17-OHP, ACTH, androstenedione and testosterone. Plasma renin activity was measured in patients with the SW form of the disease. After onset of puberty, measurement of follicle-stimulating hormone (FSH) and luteinizing hormone (LH) was added to the evaluation. FSH, LH, ACTH, androstenedione and testosterone were analyzed with chemiluminescence (IMMULITE 2000, SIEMENS), whereas17-OHP was analyzed with ELISA (Chemwell analyzer, FL, USA). The metabolic control was evaluated yearly. If the appropriate dose of hydrocortisone was prescribed, the 17-OHP, androstenedione and ACTH levels and linear growth above 10‰ on the standard growth curve were indicators of metabolic control and compliance. In pubertal boys, s.d. for predicted mid-parental height was calculated. Arbitrarily, patients who had ≥70% of 17-OHP measurements equal or below 10 ng/mL accompanied with normal ACTH levels were considered to be under satisfactory metabolic control and adherent to therapy. Comparison of the metabolic parameters, BMI and height were compared between the adherent and non-adherent group of patient.

Testicular ultrasonographic examination was introduced in 2001 and was initiated at the age of 4 years and repeated yearly. All patients were examined by the same trained doctor on the technical equipment SonoScape SSI-5000 (Providan Medical Equipment, Highland Heights, OH, USA). Longitudinal and transverse axes of the testes and TART, when visible, were measured. The ultrasonographic finding was classified in five stages as previously described (16). Magnetic resonance imaging (MRI) of the testes was performed in the first 5 patients with TART. Biopsy of the testicular tumor was performed in 2 patients who had a large tumor on ultrasonography. Slides were immunohistochemically stained with antibodies against alpha-inhibin, melanA, calretinin, synaptophysin, chromogranin, vimentin, CD56 and CD10 and analyzed on the microscope Olympus BX 41. Sperm examination was performed in two adolescents at 18 years.

### Ethical approval

Procedures performed in studies involving human participants were in accordance with the ethical standards of the institutional and/or national research committee and with the 1964 Helsinki Declaration and its later amendments or comparable ethical standards.

## Results

During the period 1991–2015, 71 Macedonian patients were diagnosed with CAH (26 with SW form, 21 with SV and 24 with non-classic (NC). In the 30 males analyzed for this study in the period 2001–2016, the following disease forms were noted: SW in 16 (53.3%), SV in 9 (30%) and NC in 5 (16.7%). From 2005, we have systematically followed 25 boys in the age group 4–18 years during a 10-year period and diagnosed eight of them (32%) with TART. Five of the patients with TART had severe SW form, two had SV form and one NC form of the disease. Molecular analysis confirmed severe mutations in six. One patient was homozygous for the p.I172N (c.518T>A) mutation, typical for the SV form. One patient was homozygous for the mild p.P30L (c.92C>T) mutation, typical for the NC form (Table 1).

**Table 1:** Clinical data, genetics and TART characteristics.

Patient	Age at diagnosis/clinical form	Ultrasound follow-up before TART (years)	Age at Dg of TART (years)	Tanner stage/tumor stage	Genotype allele 1/allele 2	Metabolic control	MRI/histology	TART after tightened control
1^a,b^	Newborn/SW	4	14.9	4/4	p.Q318X/p.Q318X	Poor	+/+	Larger
2	Newborn/SW	7	12.5	3/3	In2G/In2G	Poor	NA/NA	Smaller
3^c^	Newborn/SW	4	14	5/5	In2G/In2G	Poor	+/+	Larger
4^b^	Newborn/SW	7.5	15.3	5/2	In2G/p.Q318X	Poor	+/NA	Smaller
5	9 years/SV	5	11	2/2	p.P30L/p.P30L	Tight	NA/NA	Unchanged
6^d^	2 years/SV	5	7	1/2	p.I172N/p.I172N	Tight	+/NA	Unchanged
7	Newborn/SW	9	16	5/2	del8bp ex3/del8bp ex3	Tight	+/NA	Smaller
8	9 years/SV	7	10	1/2	In2G/In2G	Poor	NA/NA	Unchanged

^a^Patient developed mixed ALL/AML leukemia; ^b^patient has a brother with the same mutation and metabolic control, but no TART; ^c^Leydig cell tumor suspected; ^d^previously unreported genotype.

MRI, magnetic resonance imaging; NA, non-applicable; SV, simple virilizing; SW, salt wasting; TART, testicular adrenal rest tumors.

A total of 289 follow-up visits were performed prior to the diagnosis of TART (6–28 per patient). Therapy with hydrocortisone was tailored according to the 17-OHP levels. Of the 8 patients with TART, 3 were under satisfactory metabolic control. The remaining 5 were under poor metabolic control during various periods of the study. Children non-adherent to treatment in both groups with and without TART had higher 17OHP and androstenedione levels, whereas no significant difference in the height, BMI or onset of puberty was revealed (Table 2). When we compared clinical and laboratory values between children with and with no TART, these significances were lost (Table 3). Thus, all analyzed data were similar in children with and without TART.

**Table 2:** A comparison of the clinical and biochemical parameters of CAH patients adherent and non-adherent to treatment both who did or did not developed TART at the time of the last scrotal ultrasound examination (mean ± s.d. or median and range).

	Adherent		TART + no TART	Non-adherent		TART + no TART	*P*
	TART (3/8)	No TART (6/17)	9/25	TART (5/8)	No TART (11/17)	16/25	
Age at evaluation (years)	14.1 ± 1.3	16.2 ± 1.7	15.7 ± 2.6	16.5 ± 2.3	14.6 ± 3.1	15.0 ± 2.7	NS
17OHP (ng/dL) (0.2–2.3)	9.7 ± 2.2	11.1 ± 5.2	10.3 ± 4.1	19.3 ± 1.9	16.3 ± 2.3	17.6 ± 3.4	<0.001
Androstenedione (ng/mL) (0.6–2.7)	16.2 ± 4.3	12.4 ± 4.1	14.2 ± 2.6	20.2 ± 2.3	18.8 ± 4.7	19.6 ± 3.9	<0.01
Testosterone (nmol/L) (8.5–55.5)	32.0 ± 15.3	26.6 ± 15.2	29.8 ± 21.1	30.0 ± 14.2	28.1 ± 12.2	29.1 ± 13.6	NS
Height (SDS)	−1.1 (−0.9/−1.3)	−1.2 (−1.1/1.3)	−1.1 (−0.8/−1.3)	−1.3 (−1.2/−1.4)	−1.3 (−1.0/−1.6)	−1.3 (−1.0/−1.6)	NS
BMI (SDS)	0.42 ± 0.2	0.35 ± 0.6	0.39 ± 0.5	0.52 ± 0.9	0.47 ± 1.3	0.49 ± 1.2	NS
Age at puberty (years)*	12.2 (11.9–13.1)	12.7 (11.6–12.9)	12.5 (11.6–12.0)	11.5 (11.0–12.5)	12.3 (11.6–12.8)	12.1 (11–12.8)	NS

*Puberty was assessed by initial increase of the testicular volume presented as mean and range in brackets.

17OHP, 17OH progesterone; NS, non-significant, normal values are given in brackets; SDS, standard deviation score, TART, testicular adrenal rest tumor.

**Table 3:** A comparison of clinical and biochemical data in patients with and without TART (mean ± s.d. or median and range).

	TART (8/25)	No TART (17/25)	*P*
Age at evaluation (years)	15.3 ± 1.4	15.5 ± 2.7	
17OHP (ng/dL) (0.2–2.3)	15.1 ± 3.2	13.2 ± 3.5	NS
Androstenedione (ng/mL) (0.6–2.7)	18.2 ± 3.8	16.3 ± 3.2	NS
Testosterone (nmol/L) (8.5–55.5)	31.0 ± 14.9	27.5 ± 13.2	NS
Height SDS (range)	−1.1 (−0.8/−1.4)	−1.3 (−1.1/−1.6)	NS
BMI SDS (range)	+0.49 (+0.35/+0.55)	+0.41 (+0.22/+0.63)	NS
Age at puberty (years) (range)	11.7 (11.1/12.2)	12.5 (11.5/13.0)	NS

The ratio of patients under good metabolic control was similar in the group without TART (6/17 vs 3/8) (*P* = 0.17). It should be noted that two boys with TART (both non-compliant) each had a non-compliant brother with the same *CYP21A2* mutation, but neither of them had TART. One patient had large, palpable tumors on both testes; he had been consistently non-compliant for the prescribed therapy (Table 1). FSH, LH and testosterone values during the follow-up were different depending on the stage of puberty (data not shown).

**Figure 1:**
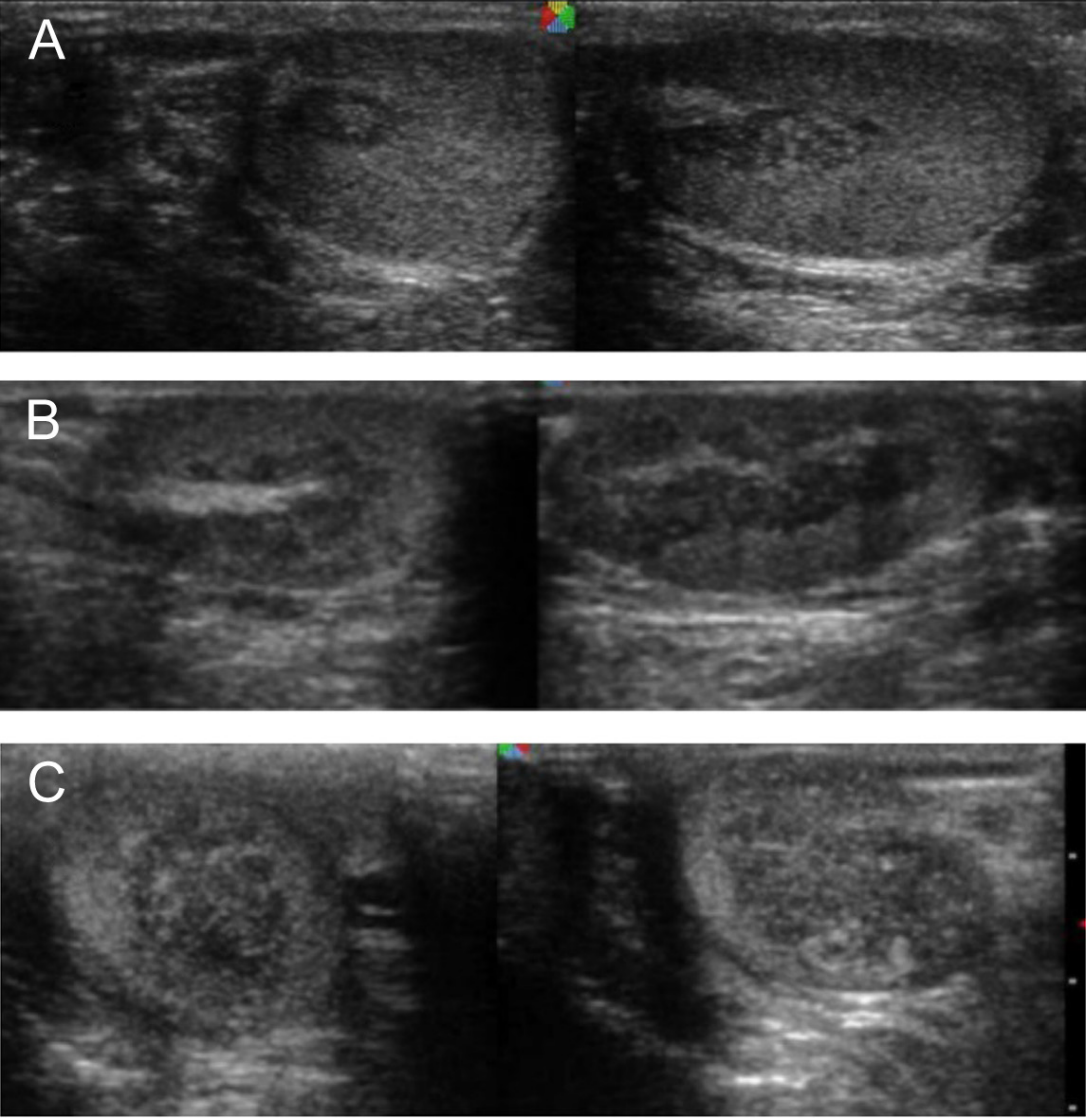
Testicular ultrasonography in patients showing TART location around testicular hilus. (A) Patient 6. (B) Patient 2. (C) Patient 7.

**Figure 2:**
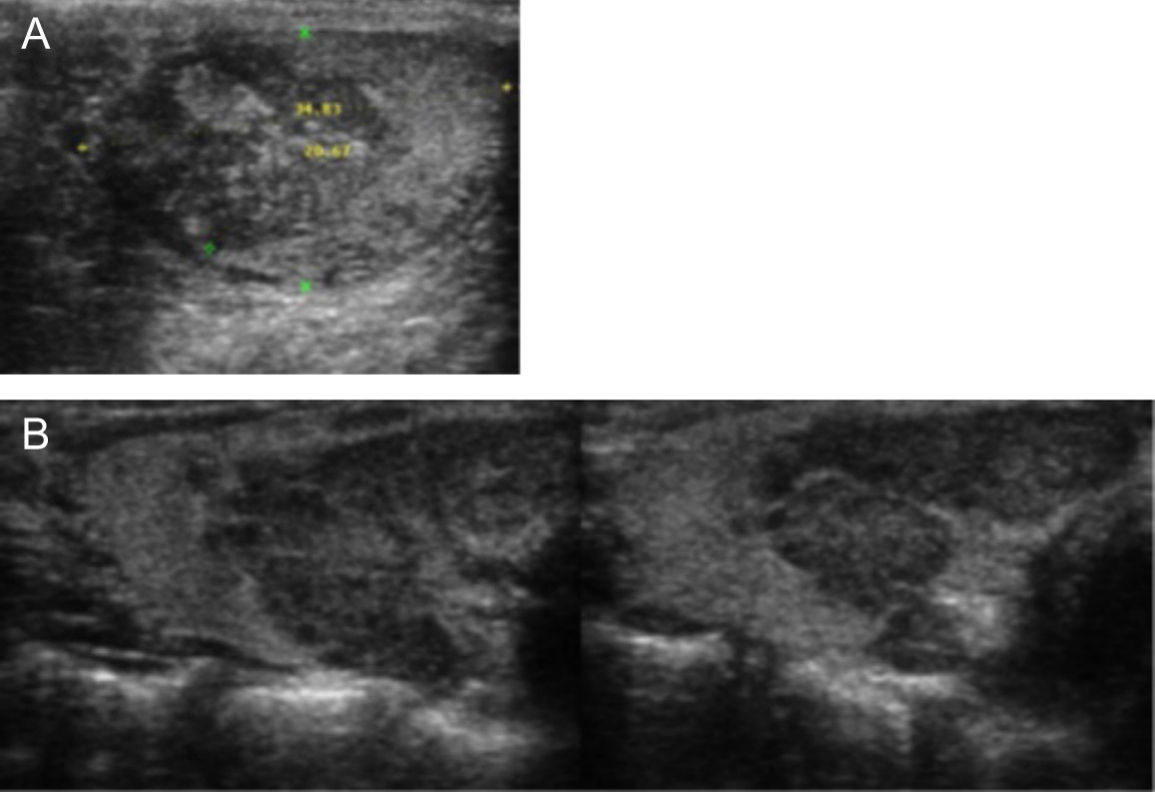
Patient 1 with poor metabolic control and evolution of TART within 4 years of follow-up. (A) Changes in the testicular tissue at the detection of TART. (B) TART after 4 years of poor metabolic control.

A total of 123 yearly testicular ultrasonography examinations (1–10 per patient) were performed during the study period, out of which 39 in the patients with TART. The children who reached adolescence during this period were followed up until the age of 21 years when they were referred to an adult endocrinologist. In the three youngest patients with early ultrasonographic examination at the ages of 7, 10 and 11 years, respectively, the tumor size was 4–10 mm in diameter, whereas in the older patients (aged 12–18 years) the size reached 12–35 mm. TARTs were localized mostly around the rete testis (Fig. 1). When TART was detected, we continued to follow-up patients at 2-month intervals in order to keep the 17-OHP values within the appropriate levels. In 6 patients whose 17-OHP levels improved, TART decreased or did not progress during 2–4 years of follow-up (Table 1). However, in two other patients, the tumor increased despite the changes in dose of hydrocortisone and improved metabolic control (Fig. 2).

**Figure 3:**
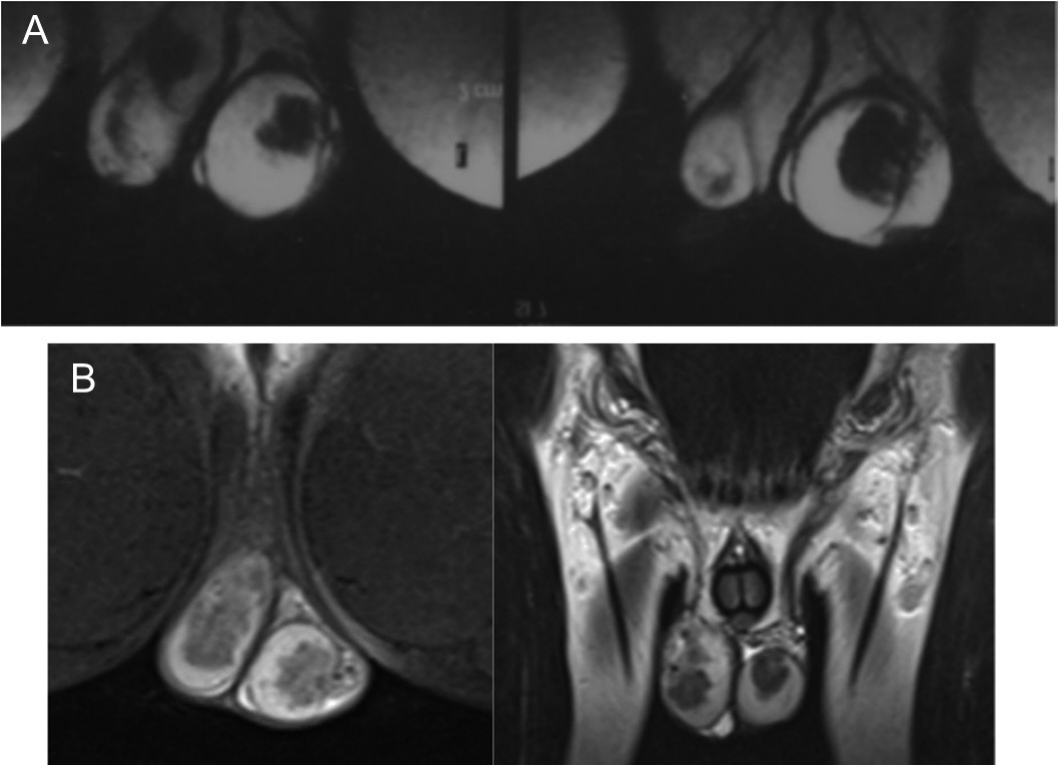
Testicular MRI in patients with TART. (A) Patient 1 during the follow-up, TART is spread around the testicular hilus. (B) Patient 3 with a large TART involving the entire testis. This patient underwent orchiectomy due to the significant discomfort.

**Figure 4:**
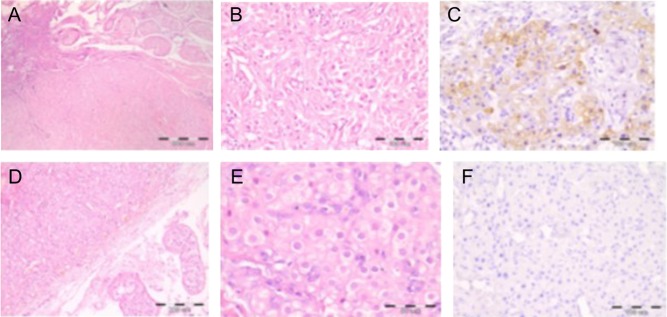
Histology of the testes in the two biopsied patients. Upper line: Patient 1. (A) The arrow points to the delineation between adrenal and testicular tissue. (B) Larger view. (C) Positive inhibin staining. Lower line: Patient 3. (D) Adrenal tissue in the testis (magnification ×400). (E) Closer image of the TART tissue. (F) Negative inhibin staining.

MRI was performed in 5 boys with TART detected on ultrasound. It confirmed the distribution of the TARTs around the hilus of the testis in all of the patients (Fig. 3). However, since no significant contribution to the diagnosis was achieved, we did not perform it in the remaining boys. In two boys with large TARTs testicular biopsy was performed. In one of them (patient 3), Leydig cell tumor was considered histologically and additional staining methods and follow-up were required to confirm TART. Microscopically, the testicular core biopsy of the two patients showed similar morphological features. Sheets and nests of uniform large, polygonal cells with abundant eosinophilic cytoplasm and round central nuclei were found. Some of the cells contained lipochrome pigment. There was mild nuclear pleomorphism without mitotic activity. The sheets and nests were separated by thin fibrous septa. Reinke’s crystals were not found. Both cases showed positive diffuse or patchy staining for alpha-inhibin, melanA, calretinin, CD56, synaptophysin and chromogranin, consistent with TART (Fig. 4). Sperm examination showed normal semen volume with decreased sperm concentration in two boys at 18 years of age.

One severely non-compliant adolescent with TART developed mixed lymphoblastic/myeloblastic leukemia at the age of 16 years, resistant to therapy and with lethal outcome within one year.

## Discussion

TARTs in male patients with CAH have been analyzed in many studies and the quest for risk factors for this complication is still not complete (11, 13, 16, 17). TART has been found in 30–94% of adults with CAH (4, 11, 12, 18, 19) using testicular ultrasound. Although substantial evidence suggests that TART is frequently present in adolescence, studies in children searching for the first symptoms of TART are of more recent dates, the prevalence of TART in children being in the range of 18.3–48% (8, 20). TART has been confirmed in a child as young as 4 years (20), 6 years and 7.5 years (16), but it is rare before adolescence. Systematic ultrasonographic search for TART in children has rarely been reported in the literature. By yearly ultrasonographic examinations during a 15-year period, we found TART in 8 out of 25 boys with CAH (32%), three of them very young, aged 6, 10 and 11 years, respectively.

In patients with CAH therapeutic non-compliance or inadequate treatment can contribute to TART development (9, 10, 19). High levels of ACTH might start the process of adrenal cell stimulation in the testes even prenatally (12, 16).

Difficulties to maintain good hormonal control are frequent in patients with CAH, especially during puberty due to hormonal changes influencing the adrenal androgens, but also due to pubertal psychosocial issues and non-compliance (16, 17, 21). Temporary changes in the metabolic control are more often the rule than the exception. Steady continuous metabolic control is found in only one-third of patients (21, 22). In a study of 11 boys with TART, Aycan *et al*. detected only 2 patients under tight metabolic control, but they did not describe the control in the other 49 boys without TART (20). Finkelstein *et al*. followed 244 patients with CAH and concluded that inappropriate control and adverse outcomes including TART are common (33% of boys and 44% of men with CAH) (18). However, TART can appear in well-controlled and even over-treated patients with CAH (13, 23). In our study, three out of 8 patients with TART were well controlled. However, only 6 out of 17 patients without TART were under tight metabolic control in more than 75% of visits. The reason for worsening of the metabolic parameters is not always easy to assess because of the variable therapeutic compliance. The parents of Patient 3 found a large number of hidden hydrocortisone tablets during home relocation. On the other hand, in our study, two pairs of brothers with the SW form of CAH were under similar metabolic control, but only one brother developed TART. Therefore, the compliance and the tight metabolic control is not the only factor contributing to TART (11, 17). Our findings are comparable with those of Stikkelbroeck *et al*. who described 11 patients with adequate treatment and high compliance, all of which developed TART (17). Most of the published studies indicate that TART is associated only with classical forms of the disease, e.g. a null group carrying null mutations with no enzyme activity, a group containing In2G mutations with negligible enzyme activity, or group B composed of patients with a homozygous p.I172N mutation causing the SV form of the disease with enzyme activity up to 2% (6, 20, 14, 17). However, isolated cases with 11-hydroxylase and 3b-hydroxyl steroid dehydrogenase gene mutations as well as rare cases with non-classical phenotypes have also been reported (6, 24). Thus, all genetic groups of CAH can develop TART although with varying incidence. Our patients belonged to group A (five with SW form) and B (two with SV form). One of the patients was homozygous for p.P30L. This mutation is considered mild, however, when homozygous, can cause increased virilization similar to the SV form (25, 26, 27). We have not found a patient with TART and this genotype in the literature. Having in mind that the genotype–phenotype correlation in CAH is not found in all patients (2, 28), genetic analysis of larger TART patient cohorts might discover additional genotypes that are associated with TART.

Ultrasound examination is very useful in the detection of the initial changes in the testes, staging and for the subsequent follow-up of therapy (8, 11, 23). All our patients with TART showed changes in the structure during the follow-up. Timely detection of TART is important in order to adjust therapy and prevent long-term damage of the functional testicular tissue (14, 17). Spermatogenesis is affected by TART depending on the stage and extension of the testicular damage (5, 16, 20, 29). In the large study of 164 men with CAH, 34% of whom had TART, Bouvatier *et al*. found significantly prevalent azoospermia in these patients compared with those who did not develop TART (4). According to Falhammar *et al*. patients with TART are less likely to father biological children (30). Interestingly, patients with the p.I172N genotype have been reported to have the most severe infertility due to lower sperm quality compared to patients with other severe mutations (27, 30). This phenomenon has not been thus far elucidated. The latest large multicenter study confirmed significant fertility impairment in adult males with CAH both presenting TART, and without TART suggesting that fertility should be kept in mind in all male children with CAH since the diagnosis (31).

MRI was used in some studies and isolated patients with TART. It can be helpful in confirming the initial lesions, however, most of the authors prefer the ultrasound due to the common availability and price (12, 13, 16). We have performed it in 5 of our patients who were positive on ultrasound and it only confirmed the ultrasound findings; therefore, we would not recommend it as a routine analysis in TART. Most endocrinologists do not recommend biopsy if the other criteria (clinical, ultrasonographic, MRI) for TART are met. However, sometimes testicular biopsy is needed for histological differentiation and excluding Leydig cell tumor (32, 33). Usually the TART biopsy shows adrenal histology adjacent to the testicular tissue, as was the case in our patients. Rarely, TART can be associated with Leydig cell tumor (24, 34). In two of our patients, we performed testicular biopsy due to the size of the tumor and predominance in one of the testicles. One of them was initially diagnosed as Leydig cell tumor; however, the specific staining and the evolution excluded this diagnosis. The other boy had a peculiar disease course, with many challenges in the metabolic control. The biopsy confirmed TART. However, at the age of 16 years, he developed rapid progressing and therapy-resistant mixed lymphocytic/myeloblastic leukemia and died within a year of the diagnosis. To our knowledge, a similar case where TART was associated with malignancy has not been described so far and their relation remains elusive.

Several studies have shown improvements using increased doses of hydrocortisone and fludrocortisone (22, 35, 36). While some authors refer to complete remission of the tumors after increased doses of hydrocortisone, others find temporary improvement in some patients, and no response in others (17, 20). In our patients, increased doses showed slight decrease of the tumor size in 3 patients and stabilization in another 3; there was no improvement in the remaining 2. Side effects of higher steroid doses such as hypertension, obesity and insulin resistance have been described (22, 30, 37). When no improvement with hormonal adjustment is achieved, testis sparing surgery is warranted (33, 38). However, TART might reappear after surgery. Preservation of sperm in young adults should also be considered while the sperm quality is still satisfactory (35).

The strengths of our study are the relatively large number of young boys with CAH followed systematically by yearly ultrasound in the same center for 15 years. The weakness is the small number of patients due to the rarity of the disease and arbitrary judgment of the metabolic control as a percentage of adequate 17OHP levels having in mind that some of these boys have periods of better and worse control.

In conclusion, the treatment of classical forms of CAH in young boys is generally suboptimal, not strictly dependent on the *CYPA2* mutation, and can deteriorate towards TART development around puberty. However, appearance at a younger age and in well-controlled patients shows that the continuous suboptimal control is not the single reason for TART. The contribution of the treatment, the severity of the mutation or other unknown factors remains to be elucidated. Early detection and follow-up of TART by ultrasound is useful for the preservation of testicular function.

## Declaration of interest

The authors declare that there is no conflict of interest that could be perceived as prejudicing the impartiality of the research reported.

## Funding

This research did not receive any specific grant from any funding agency in the public, commercial or not-for-profit sector.

## Author contribution statement

M K diagnosed, treated, followed the patients, designed, wrote and edited the manuscript; V J participated in the writing and editing and performed histology of testicular tissue; V A performed and discussed genetic analyses and participated in the writing and editing of the manuscript.

## Informed consent

Informed consent was obtained from all individual participants included in this study.
